# Effect of Sonication Duration in the Performance of Polyvinyl Alcohol/Chitosan Bilayer Films and Their Effect on Strawberry Preservation

**DOI:** 10.3390/molecules24071408

**Published:** 2019-04-10

**Authors:** Jie Ding, Rong Zhang, Saeed Ahmed, Yaowen Liu, Wen Qin

**Affiliations:** 1 College of Food Science, Sichuan Agricultural University, Yaan 625014, China; dingjiedream@163.com (J.D.); zhangronglzy@163.com (R.Z.); saeedahmed_mahar@yahoo.com (S.A.); 2 College of Food Science and Technology, Sichuan Tourism University, Chengdu 610100, China; 3 School of Materials Science and Engineering, Southwest Jiaotong University, Chengdu 610031, China; 4 California NanoSystems Institute, University of California, Los Angeles, CA 90095, USA

**Keywords:** polyvinyl alcohol, chitosan, bilayer films, strawberry preservation

## Abstract

In this study, we fabricated polyvinyl alcohol (PVA)/chitosan (CS) bilayer films by casting and investigated the effects of preparation conditions and CS content (2, 2.5, or 3 wt.%) on the ability of these films to preserve packaged strawberries. The best performance was achieved at a CS loading of 2.5 wt.% (ultrasound time, 25 min); the strain and stress values were 143.15 ± 6.43% and 70.67 ± 0.85 MPa, respectively, oxygen permeability was 0.16 ± 0.08 cm^2^·m^2^·day^−1^·MPa^−1^, water vapor permeability was 14.93 ± 4.09 g·cm^−1^·s^−1^·Pa^−1^, and the shelf life of fresh strawberries packaged in the PVA/CS 2.5 wt.% bilayer film was determined to be 21 days at 5 ± 2 °C and a relative humidity of 60 ± 5%. Treatment with PVA/CS bilayer films prevented the decrease in the firmness of strawberries during storage (21 days). The evaluated physicochemical parameters (weight loss, decay, firmness, titratable acidity, soluble solid content, ascorbic acid content, and color) indicated that treatment with PVA/CS bilayer films led to better maintenance of the fruit quality. We believe that our study makes a significant contribution to literature because it paves the way to the fabrication of smart packaging materials and facilitates the commercialization of fresh strawberries as an important health food.

## 1. Introduction

Strawberries, which are a highly popular non-climacteric summer fruit rich in polyphenols, anthocyanins, vitamins, and amino acids, should be harvested when fully mature to achieve maximum quality [1]. However, the lack of a protective rind makes strawberries highly susceptible to fungal decay, mechanical injury, water loss, and physiological deterioration during storage, thereby leading to a short shelf life [2]. In particular, the main pathogen attacking strawberries during storage is *Botrytis cinereal*, a saprophytic fungus also known as gray mold [3]. This fungus predominantly infects strawberries during cultivation and can easily spread to nearby fruit, while symptoms mainly develop only after harvesting. To avoid this infection, the canopy of strawberry plants is usually sprayed with fungicides several times per season [4]. However, strict normative restrictions and the growing concern of consumers regarding fungicide residues limit the use of conventional fungicides, thus necessitating the search for better preservation alternatives.

To slow down the metabolism and reduce the extent of deterioration during storage or transport, strawberries are cooled immediately after being harvested [5]. In addition, the postharvest lifetime of strawberries can be further extended by combining refrigeration with other techniques such as the use of a modified atmosphere, as confirmed by studies on the storage-induced degradation of soluble sugars, ascorbic acid, and other acids [6]. The high potential of the atmosphere modification technique and the growing interest of large-scale retail trade in strawberries drew increased attention to the development of novel food packaging techniques, particularly those avoiding environmental pollution with plastics via the use of bio-based polymers [7]. Polyvinyl alcohol (PVA) is a non-toxic, biocompatible, and water-soluble semi-crystalline polymer featuring unique physical properties arising from the presence of numerous –OH groups and hydrogen-bond formation [8]. Moreover, PVA exhibits an excellent film-forming ability and is consequently one of the best bio-based polymers for packaging.

Natural antimicrobial agents exhibit the advantages of non-toxicity, biodegradability, and wide availability and are, therefore, ideally suited for fruit treatment [9,10]. One such agent is chitosan (CS), a cationic amino polysaccharide (product of chitin deacetylation) that can inhibit the growth of many pathogenic bacteria and fungi [10]. Consequently, CS is widely used to protect strawberries from pathogenic infections, and it acts as a mechanical barrier to the spread of such infections during storage [11]. El Ghaouth et al. demonstrated that the antibacterial activity of CS may be ascribed to the fact that this compound activates chitinase, a defense enzyme that catalyzes the hydrolysis of chitin, an important component of fungal cell walls, thus preventing the growth of fungi on fruit [12]. According to previous studies, CS preserves the freshness of fruit by both directly interfering with fungal growth and by activating several biological processes in plant tissues [13].

Although PVA/CS bilayer films are known to exhibit good physical properties and antibacterial activity owing to specific intermolecular interactions between PVA and CS, their use to preserve strawberries is not extensively reported. Therefore, in this work, we aimed to study the effect of the CS content (2, 2.5, and 3 wt.%) in PVA/CS bilayer films on the efficiency of strawberry preservation at 20 °C. Firstly, an optimal ultrasonic time was selected, leading to optimal viscosity of the solution and the mechanical properties of the two-layer membrane were analyzed; that is, the effects of different ultrasonic times on the film-forming properties of the bilayer films were investigated. Then, we characterized the water vapor permeability (WVP) and oxygen permeability (OP) of the fabricated films. Non-packaged and packaged strawberries were subjected to sensory evaluation and compared/analyzed in terms of their color, firmness, soluble solid content (SSC), titratable acidity (TA), weight loss, and percentage of decay (% decay). Additionally, we also probed the effect of the storage temperature on the shelf life and % decay.

## 2. Materials and Methods

### 2.1. Materials

Low-viscosity chitosan (food grade, deacetylation degree = 90%, molecular weight (Mw) = 165,000 Da) was obtained from Shandong Aokang Biological Technology (Shandong, China). PVA (98%; Mw = 124,000–186,000 Da, hydrolysis degree = 87–89%) was purchased from Sigma Aldrich (St. Louis, MO, USA). Acetic acid was obtained from Guoling Chemical Reagents Co. (Chengdu, China) unless otherwise indicated. Fresh strawberries of commercial maturity were harvested at a local farm in Shuangliu (Chengdu, China), transported to the laboratory in a cooled state, and used for packaging experiments on the same day. Defective fruit units, i.e., those showing signs of decay and mechanical damage, were eliminated, and strawberries of uniform size, color, and shape were consequently used in the above experiments.

### 2.2. Preparation of CS Solutions by Ultrasonication

CS was dissolved in 2 vol.% aqueous acetic acid to concentrations of 2, 2.5, or 3 wt.% by stirring overnight, and then degassed in a vacuum desiccator. Subsequently, the solutions were split into six portions, each of which was ultrasonicated (JY98-IIID; Ningbo Scientz Biotech Co., Ltd., Ningbo, China) in a water bath (80 °C) for 5, 10, 15, 20, 25, or 30 min at an output power of 15,000 W; the obtained samples were denoted as X-m, where X is the solution type, and m is the ultrasonication time. The viscosity of the colloid suspension obtained after ultrasonication was measured using a Brookfield viscometer (Model DV-II + Pro, Middleboro, MA, USA). All tests were carried out in triplicate, and the results are presented as average values.

### 2.3. PVA/CS Bilayer Films Preparation

A 10 wt.% PVA solution in distilled water (prepared at 100 °C, stirring for 10 h) was cast on a 30-cm^2^ solute glass plate and dried at 60 °C for 24 h to obtain a PVA film with uniform thickness. Further, the final ultrasonicated CS solutions were cast onto the PVA film using a coating machine to afford PVA/CS bilayer films, which were sequentially washed with 2 wt.% aqueous NaOH (to remove residual acid) and then distilled water, before finally being dried in a desiccator at room temperature (25 °C) for 24 h to achieve a uniform thickness of 3 mm.

### 2.4. Fruit Preparation and Packaging

Strawberries were randomly divided into five groups of 84 strawberries. Four groups were used for PVA (PVA), PVA/CS 2 wt.% (2%), PVA/CS 2.5 wt.% (2.5%), and PVA/CS 3 wt.% (3%) packaging treatments, and the fifth group contained control (unpackaged) strawberries. Each packaging experiment was conducted in duplicate, and the remaining strawberries were used for sensory evaluation tests. Packaged samples were placed in plastic boxes and stored at 7 °C with relative humidities (RHs) of 70–75%. Packaged and control samples were evaluated after 3, 6, 9, 12, 15, 18, and 21 days of storage, while the latter were also evaluated at the beginning of the experiment (i.e., on day zero).

### 2.5. Film Characterization

Attenuated total reflectance Fourier-transform infrared (ATR-FTIR) spectrometry (ATR-FTIR, Nicolet 5700, Thermo Nicolet Instrument Corp., Madison, WI, USA) was used to identify the chemical structures of the PVA/CS bilayer films and the possible interactions between their components. A small section cut from each composite film was used. The samples were analyzed at a resolution of 4 cm^−1^, with an aperture setting of 6 mm, scanner velocity of 2.2 kHz, background scan time of 32 s, sample scan time of 32 s, and a total of 100 scans per sample, in the range of 4000 to 650 cm^−1^. Film thicknesses were measured by sampling at 10 random positions with a ZUS-4 micrometer (Yue Ming Small Machine Co., Ltd., Changchun, China), and the average values were calculated from these measurements; these values were subsequently employed for OP and mechanical property modeling.

Mechanical properties of ten 20 × 150 mm film strips were evaluated using an automatic tensile tester (Shanghai Super Technology Instrument Co., Ltd., Shanghai, China, following ASTM D882-18—method for tensile properties of thin plastic sheeting [14]). Force (N) and deformation were recorded during the extension of the film strips at 300 mm/m with an initial distance of 50 mm between film-clamping grips [8]. Tensile strength (MPa) was calculated by dividing the maximum load by the initial cross-sectional area of the sample. Elongation at break (%) was calculated as the ratio of the strip length at the point of failure to the initial length. OP tests (PERME TM OX2/231 permeability tester; Labthink Instruments Co., Ltd., Jinan, China) were performed using oxygen as the test gas (RH ≈ 50%; flow rate = 20 mL·min^−1^) and nitrogen as a carrier gas (10 mL·min^−1^) at 23 °C (following ASTM D1434—standard test method for determining gas permeability characteristics of plastic film and sheeting [15]). WVP values were determined according to ASTM E96-95 using a Perme VAC-V1 instrument (Labthink, Jinan, China, following the ASTM E96-95 gravimetric method [16]). The WVP (g·cm^−1^·s^−1^·Pa^−1^) was calculated as follows:

WVP = WVT × T/(P × (R_1_ − R_2_))

where T is the average thickness of the film, P is the saturation vapor pressure of water at the test temperature (25 °C), R_1_ is the relative humidity in the desiccator, and R_2_ is the relative humidity inside the special aluminum cup. Three specimens per film were tested.

### 2.6. Quality Evaluation After Storage

The quality of strawberries was evaluated every three days for a total of 21 days. At least five separate experiments were performed to collect each datum. Error bars are used to indicate the estimated error in a measurement or the uncertainty in a value.

#### 2.6.1. Weight Loss Measurements

Weight loss during storage was measured daily using an FA 2004 W balance (±0.0001 g; Shanghai Jinghai Instrument Co., Ltd., Shanghai, China) and expressed as (*W*_0_ − *W*_t_)/*W*_0_, where *W*_0_ and *W*_t_ are the initial weight of the strawberry and its weight after time *t*, respectively.

#### 2.6.2. Evaluation of the Degree of Decay

The decay of strawberry was characterized every day according to a five-point empirical scale (Table 1) [17].

Both the incidence and severity of decay are reported as weighted mean percentages of the maximum possible level; they were calculated using the equation proposed by McKinney [18].

(1) $$I=\left[\sum \frac{\left(d\times f\right)}{\left(N\times D\right)}\right]\times 100\%$$

where *I* is the category of rot intensity scored on fruit, *f* is rot occurrence frequency, *N* is the total number of examined fruit (both healthy and infected), and *D* is the highest category of decay intensity observed on the empirical scale.

#### 2.6.3. Color Measurements

Color was evaluated using a CS-10 spectrocolorimeter (Color Spectrum Technology Co., Ltd., Hangzhou, China) calibrated with a standard white-and-black tile. Measurements were carried out in the same previously marked area to avoid the effects of fruit surface heterogeneity [8]. Luminosity (L^*^) represents the darkness of black and the brightness of white, with the darkest black corresponding to L^*^ = 0 and the brightest white corresponding to L^*^ = 100. Chroma (C^*^_ab_) was calculated as

(2) $$C{*}_{ab}=\sqrt{a^{*2}+b^{*2}}$$

and hue angle (h^*^_ab_) was calculated as

(3) $$h{*}_{ab}=\tan^{-1}\left(b^{*}|a^{*}\right)$$

where *a* and *b* are the color parameters of the fruit.

#### 2.6.4. Firmness Measurements

Firmness was evaluated at room temperature using a texture analyzer (Shanghai Supertechnical Instruments Co., Ltd., Shanghai, China) equipped with a 5-kg load cell. A single whole strawberry was placed on a flat platform and penetrated with a 2-mm-diameter flat-head stainless-steel cylindrical probe to a depth of 6 mm at a speed of 1 mm·s^−1^. After firmness measurements, strawberries were cut into small pieces and homogenized by blending for 2 min at high speed in a hand-held blender. The homogenized strawberry puree was divided into 5-g, 5-g, 10-g, and 5-g portions with the strawberry juice [19].

#### 2.6.5. TA Measurements

Homogenized strawberry puree (5 g) was suspended in hot distilled water (50 mL, 80 °C) for 30 min. The suspension was filtered, and the filtrate was titrated to pH 8.1 with 0.01 M NaOH. The TA of the diluted puree (10 g citric acid·kg^−1^ dry weight) was calculated as

(4) $$\mathrm{TA}\;=\frac{V_{\left(\mathrm{NaOH}\right)\;}\times \;0.01\;\times \;0.064\;\times \;V_{0}}{m\;\times \;V_{1}}$$

where *V*_(NaOH)_ (mL) is the volume of the 0.01 M NaOH solution, 0.064 is the conversion factor for citric acid, *V*_0_ is the volume of the volumetric flask, *m* is the quality of the homogenized strawberry puree, and *V*_1_ is the filtrate volume [8].

#### 2.6.6. SSC and pH Measurements

SSC and pH values were determined for 5-g strawberry juice samples using a hand-held refractometer (LYT-380, Shanghai Linyu Co., Ltd., Shanghai, China) and a pH meter (pHS-25, Chengdu Ark Technology, Co., Ltd., Chengdu, China), respectively.

#### 2.6.7. Ascorbic Acid Content Measurements

A 10-g strawberry sample was extracted with aqueous trichloroacetic acid (100 mL, 50 g·L^−1^) for 10 min, and the mixture was centrifuged to afford a clear supernatant. A 1.0-mL aliquot of this supernatant was transferred into a test tube and treated with aqueous trichloroacetic acid (1.0 mL, 50 g·L^−1^), ethanolic phenanthrolin (1 mL, 5 g·L^−1^), and ethanolic FeCl_3_ (0.5 mL, 0.3 g·L^−1^). The absorption of the obtained mixture was recorded at 534 nm against a blank sample using an absorption spectrophotometer (MAPADA Instrument Co., Ltd., Shanghai, China), and the content of ascorbic acid was estimated from a standard curve prepared using pure ascorbic acid.

#### 2.6.8. Effect of Storage Temperature on Strawberry Quality

Strawberries were randomly divided into five groups with seven replicates, packaged in PVA/CS 2.5 wt.% bilayer film, and stored at 273, 280, 287, 294, and 301 K and RH 90 ± 5%. The decay of samples stored at 301 K was characterized every two days, while samples stored at 287 and 294 K were characterized every three days, and those stored at 273 and 280 K were assessed every four days [20].

#### 2.6.9. Mathematical Modeling

Experimental data representing decay were statistically analyzed using the nonlinear regression technique of Origin 8.0 software (Northampton, MA, USA). Correlations between predicted and measured values were established in terms of the *R*^2^ coefficient and relative prediction error (RE). In particular, RE was calculated as

(5) $$\mathrm{RE}=\frac{\mathrm{Root}\;\mathrm{Mean}\;\mathrm{Squared}\;\mathrm{Error}\;\left(\mathrm{RMSE}\right)}{\mathrm{Average}\;\mathrm{of}\;\mathrm{Measured}\;\mathrm{Samples}}\times 100\%$$

(6) $$\mathrm{RMSE}=\sqrt{\frac{\sum_{i=1}^{n}{\left(Ve-Vp\right)}^{2}}{n}}$$

where *V*_e_ is the measured value, *V*_p_ is the predicted value, and *n* is the number of samples [21].

### 2.7. Statistical Analysis

Multiple samples were tested, and the results are reported as means ± standard deviation. The obtained values were subjected to analysis of variance, and the means were separated by Duncan’s multiple range test (Super ANOVA, Abacus Concepts, Inc., Berkeley, CA, USA), with *p*-values < 0.05 considered significant.

## 3. Results and Discussion

### 3.1. Effect of Ultrasonic Treatment on the Viscosity of CS Solution

Table 2 lists viscosities of CS solutions obtained after different ultrasonication times, revealing that viscosity decreased with increasing ultrasonication time and increased with increasing CS content. For example, PVA and PVA/CS 3 wt.% dispersions sonicated for 5 min featured viscosities of 3420 ± 35.51 and 10,626.67 ± 35.31 mPa·s, respectively, whereas significantly higher values (2386.67 ± 45.57 and 9440 ± 74.35 mPa·s, respectively) were obtained after 10-min sonication. After 15-min treatment, the viscosities of PVA/CS 2 wt.% and PVA/CS 3 wt.% dispersions significantly decreased compared to those of other samples. Comparing PVA/CS 2.5 wt.% (5 min) to PVA/CS 2.5 wt.% (10 min) and PVA/CS 2.5 wt.% (15 min) to PVA/CS 2.5 wt.% (20 min), the viscosity data of PVA/CS 2.5 wt.% (10 min) to PVA/CS 2.5 wt.% (15 min) decreased significantly. This result indicated that a >15 min ultrasonic process is more effective for the PVA/CS 2.5 wt.% solution. Furthermore, after 30 min of treatment, the viscosity data showed a slight increase. The above results clearly indicated that the viscosity of PVA/CS dispersions depends on the duration of ultrasonication, which may be ascribed to the effect of acoustic cavitation [22]. Prolonged ultrasonication probably increases the possibility of the conversion of macroscopic bubbles conversion into micro-bubbles that undergo cavitation, and the emission of heat and excited species during cavitation results in increased solution uniformity and reduces the influence of internal resistance of the solution on viscosity [7,23]. The viscosity increase observed after 30-min sonication was ascribed to the concomitantly increasing strength of hydrogen bonds between molecules [24].

### 3.2. Fourier-Transform Infrared Spectroscopy Analysis

**Figure 1:**
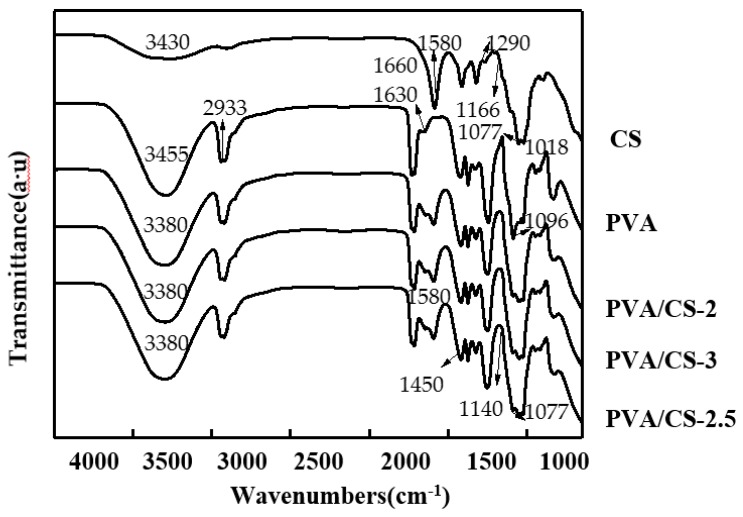
Attenuated total reflectance Fourier-transform infrared (ATR-FTIR) spectra of polyvinyl alcohol (PVA), chitosan (CS), and PVA/CS bilayer films.

Figure 1 presents attenuated total reflectance Fourier-transform infrared (ATR-FTIR) spectra of CS, PVA, and PVA/CS 2.5 wt.%. In the case of PVA, bands at 3455 and 1630 cm^−1^ were assigned to the stretching and bending vibrations of OH groups [25], respectively, while the band at 2933 cm^−1^ corresponded to the asymmetric stretching vibration of methylene groups, and that at 1096 cm^−1^ was assigned to C–O stretching in acetyl groups on the PVA backbone [26]. For CS, typical saccharide bands were observed at 1166, 1077, and 1018 cm^−1^, and peaks at 3430, 1660, and 1290 cm^−1^ were assigned to OH stretching, amide I, and amide II bands, respectively [27]. The spectra of PVA/CS 2.5 wt.% indicated a clear increase in the intensity of the band at 3380 cm^−1^, attributed to hydroxyl group stretching vibrations of PVA, with a secondary amide group of CS. The band at 1077 cm^−1^ reflected the presence of polymer backbone-bound OH and secondary amide groups. The band at 1450 cm^−1^, observed at a CS content of 2.5 wt.%, was assigned to C=N pyridine ring vibrations, which confirmed the occurrence of PVA–CS complexation [24]. The characteristic bands of all PVA/CS films resembled those of PVA, and the strong amorphous carbonyl stretching vibrations of PVA were present in all cases. With increasing CS content, the intensity of the OH peak(s) increased, and the characteristic shape of the CS spectrum changed. Moreover, peaks concomitantly shifted to lower frequencies because of the increasing strength of hydrogen bonding between the OH groups of PVA and OH/amine groups of CS in bilayer films [28]. Kim et al. reported that the intensity and position of the crystallization-sensitive band of PVA at 1140 cm^−1^ did not significantly change in PVA and bilayer films [29].

### 3.3. Mechanical Properties

Table 3 lists the mechanical properties of pure PVA and PVA/CS films, indicating that pure PVA film sustained an ultimate stress of 32.20 ± 0.28 MPa and a specific deformation of 123.23 ± 3.75%. Further, the mechanical properties continued to improve with increasing ultrasound time. For the same ultrasonic time, the ultimate stress increased, while the elongation at break decreased with increasing CS content. Bispo et al. proposed that the ultimate stress values of polymer bilayer films lie between those of pure-component films [30]. Therefore, the above results suggest that the tensile stress and maximum specific deformation of PVA/CS films are largely determined by those of PVA. An increase in the CS content resulted in stress and strain changes because of the thermodynamic immiscibility and inherent incompatibility between the thermoplastic polymer and CS [31]; moreover, the introduction of CS also disrupted the hydrogen bonds between PVA chains and affected the intermolecular hydrogen bonds formed between CS amine and hydroxyl groups [32]. However, the fact that the best mechanical properties were observed for PVA/CS 2.5 wt.% proved that PVA and CS are partially miscible [33].

### 3.4. OP and WVP

The OP of a given packaging material is an important parameter determining the food preservation performance, with low OPs indicating excellent oxygen barrier properties. Table 4 shows the OP and WVP values of the investigated films, revealing that the OP of pure PVA film (0.12 ± 0.04 cm^2^·m^−2^·atm^−1^·day^−1^·MPa) increased upon the introduction of CS, e.g., PVA/CS 2 wt.% and PVA/CS 2.5 wt.% featured OPs of 0.15 ± 0.07 and 0.16 ± 0.08 cm^2^·m^−2^·atm^−1^·day^−1^·MPa^−1^, respectively. This finding is attributed to the decreased crystallinity of films with low CS content [34]. At CS contents above 3 Wt.%, OP rapidly increased with increasing CS content, and the corresponding films consequently exhibited the worst oxygen barrier properties.

PVA/CS 2 wt.% and PVA/CS 2.5 wt.% films exhibited WVPs of 12.43 ± 3.72 and 14.93 ± 4.09 g·cm^−1^·s^−1^·Pa^−1^, respectively, which are slightly higher than that of the pure PVA film (10.11 ± 2.14 g·cm^−1^·s^−1^·Pa^−1^), whereas the PVA/CS 3 wt.% film exhibited a significantly higher WVP (*p* < 0.05). These results indicate that the PVA–CS interaction can increase the water vapor barrier properties, ascribed to (i) the hydrophilic nature of CS favoring the transport of water molecules through the film [35], and (ii) composite films with high CS contents having low crystallinity [36].

### 3.5. Weight Loss

**Figure 2:**
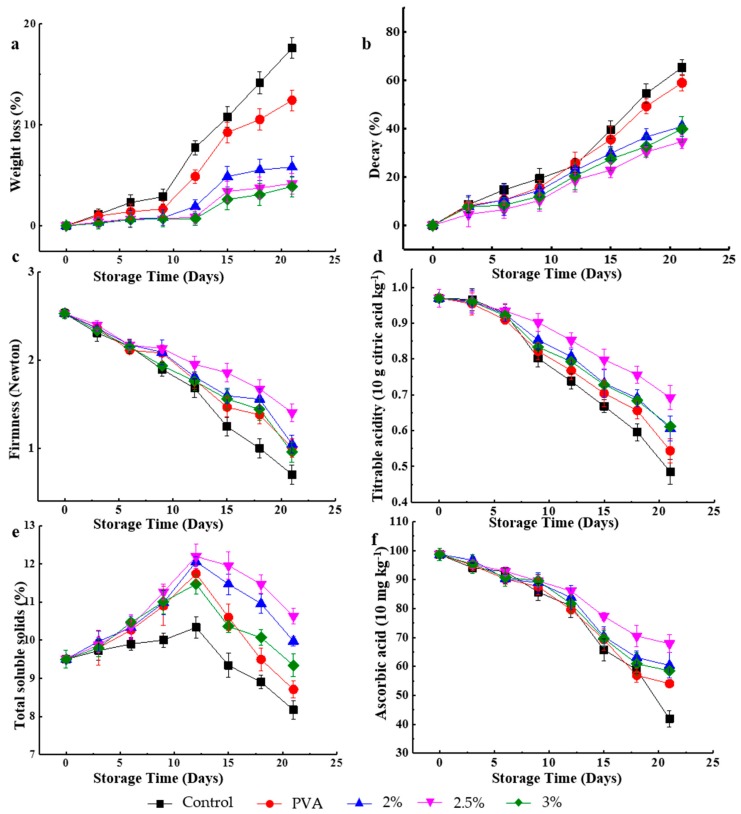
Effect of not packing and packing with PVA and different PVA/CS bilayer films on the (**a**) weight loss, (**b**) decay, (**c**) firmness, (**d**) titratable acid, (**e**) total soluble solids, and (**f**) ascorbic acid quality parameters of strawberry during various storage times (t = 6).

Weight loss can be mainly ascribed to respiration and moisture evaporation through the thin strawberry skin, resulting in fruit shrinkage and deterioration. The water loss rate was determined by (a) the water pressure gradient between the fruit and the surrounding atmosphere, and (b) storage temperature. Thus, it was concluded that strawberries should be stored at low vapor pressure differences and low temperature. Figure 2a shows the weight loss of strawberries during 21 days of cool storage, revealing that all samples exhibited a continuous weight loss with time, with the greatest loss observed for the control sample. The weight loss of this sample after nine days was significantly higher than that of packaged samples (*p* < 0.05), because it was primarily caused by the migration of water from the fruit to the environment [37]. For packaged samples, the presence of packaging materials that are semipermeable to carbon dioxide, oxygen, and moisture decreased the extent of respiration, water loss, and oxidation, which was reflected as decreased weight loss [38]. After 15 days of cool storage, the weight losses of unpackaged and PVA-packaged fruit exceeded the market limit of 15%, while this limit was not exceeded when bilayer films were used for packaging [39]. The lowest weight loss upon 21 days of storage was obtained for the PVA/CS 3 wt.% film, which is characterized by increased CS, because, as previously shown, CS can restrain the growth and reproduction of microorganisms and lower the rotting speed of cherries to some extent [40]. Thus, the PVA/CS 3 wt.% bilayer film was concluded to be best suited for cool storage of strawberries.

### 3.6. Decay

Dehydration facilitates surface damage, i.e., dehydrated strawberries are more easily infected by fungi. Figure 2b shows that although the decay percentage increased during the 21 days of storage in all cases; this increase was more significant for unpackaged and PVA-packaged samples (65.21 and 58.85%, respectively) than for PVA/CS-packaged ones (34.57–40.88%). This behavior is ascribed to the fact that PVA/CS films acted as barriers during cool storage, restricting water transfer and protecting fruit skin from mechanical injury, as well as sealing small wounds and, thus, delaying their dehydration to decrease their susceptibility to fungal infections. Some researchers stated that CS can inhibit the growth of pathogenic bacteria and fungi and acts as a mechanical barrier to protect the fruit from pathogenic infections, thereby hindering the decay during its storage [41,42]. Herein, in agreement with our previous studies, the lowest decay percentages during the whole storage period were observed when the fruit was packed in PVA/CS 2.5 wt.%, which is ascribed to the fact that this bilayer film provided an atmosphere most suited for suppressing bacterial growth [43].

### 3.7. Firmness

Firmness is one of the main parameters used to evaluate the postharvest deterioration of fruits and vegetables, since it affects their shelf life, susceptibility to pathogenic infections, transportability, and storage. During ripening, strawberries are often considerably softened because of the concomitant degradation of the middle lamella of the cortical parenchyma cell walls [44]. Moreover, firmness is also influenced by the cell wall strength, cell-to-cell contact, and cellular turgor [45]. Figure 2c shows the changes in the firmness observed during 21 days of storage of packaged and unpackaged samples, revealing that firmness values measured on the first day of cool storage (i.e., 2.525 N) are similar for control and packaged samples (*p* > 0.05) and they began to decrease after three days. The firmness of unpackaged and PVA, PVA/CS 2 wt.%, and PVA/CS 3 wt.% packaged samples significantly decreased after six (unpackaged and PVA/CS 3 wt.%) and nine (PVA and PVA/CS 2 wt.%) days of storage, while no marked decrease was observed for PVA/CS 2.5 wt.% packaged samples over the whole storage period (*p* > 0.05). After 21 days of cool storage, firmness losses of 72.3, 60.37, 58.61, 44.43, and 62.24% were observed for unpackaged, PVA, PVA/CS 2 wt.%, PVA/CS 2.5 wt.%, and PVA/CS 3 wt.% packaged strawberries, respectively, i.e., packaging helped retain the firmness and PVA/CS 2.5 wt.% proved to be the most effective. This finding is in agreement with previous reports [40], where CS and other biopolymers were shown to act as selective barriers to O_2_ and CO_2_, modifying the internal atmosphere and slowing down the respiration rate of fruit and vegetables [46].

### 3.8. Total SSC

The quality and consumer perception of fruit are strongly affected by TSS [47]. Figure 2e presents the results of TSS analysis during 21 days of cool storage, revealing that, in all cases, a significant (*p* < 0.05) TSS increase was observed for the first 12 days, which is ascribed to the occurrence of sucrose synthesis in this period [2,48]. Starting from day 13, TSS rapidly decreased in all cases, with the sharpest decrease observed for control and PVA-packaged samples (24.59 and 25.83%, respectively). This finding is attributed to the hydrolysis of sucrose to maintain physiological activity, and this hydrolysis is concluded to be most rapid for the above two samples. Moreover, bilayer-packaged samples maintained higher TSS values (with maximum observed for PVA/CS 2.5 wt.%) than those of unpackaged and PVA-packaged ones at the end of the cool storage period as a result of respiration, and CS incorporation into packaging is, therefore, concluded to prolong the shelf life of strawberries.

### 3.9. TA

After soluble sugars, organic non-volatile acids are the second most important contributors to strawberry. In strawberries, these acids are largely represented by citric acid (>90%), which regulates the cellular pH and may influence anthocyanin stability and, as a consequence, the fruit color. Nevertheless, TA changes of strawberry fruit during the cool storage are not extensively explored [49]. Respiration, resulting in organic acid consumption and fruit senescence, is thought to be one of the main reasons for TA changes [50]. Figure 2d shows the effect of packaging on strawberry TA content, demonstrating that, in all cases, TA decreased with time during cool storage, with the slowest decrease observed for fruit packaged in PVA/CS 2.5 wt.% film. This result demonstrates that packaging materials can delay the utilization of organic acids and, hence, slow the TA decrease, in agreement with our previous report [43]. In particular, the TA decrease (0.97 to 0.544 (10 g citric acid·kg^−1^) over 21 days) observed for PVA-packaged fruit was higher than that observed for PVA/CS-packaged samples, which is ascribed to the antibacterial effect of chitosan and its ability to slow down the deterioration of the fruit during cool storage. Thus, bilayer packaging materials significantly (*p* < 0.05) reduced the rate of TA decrease, with the highest TA after 21 days (0.692) being observed in the case of PVA/CS 2.5 wt.%.

### 3.10. Ascorbic Acid Content

Figure 2f displays the effect of the packaging material on the ascorbic acid content during cool storage, showing that postharvest elongation resulted in a decline of the above parameter in both packaged and unpackaged samples. After nine days of storage, the ascorbic acid content of all samples slightly decreased (*p* < 0.05), with the lowest value observed for the unpackaged sample (41.90 ± 2.83 (10 mg·kg^−1^) at day 21) and the highest value observed for the PVA/CS 2.5 wt.% packaged sample. This finding is ascribed to the ability of PVA/CS bilayer materials to reduce O_2_ diffusion, slow down the respiration rate, and thereby delay the deteriorative oxidation of ascorbic acid, in agreement with the results of previous studies [51].

### 3.11. pH

Table 5 presents pH changes in packaged and unpackaged strawberries, revealing that the sample pH increased with time in all cases. Among all packaging materials, the largest pH increase was observed for PVA, while the lowest increase was observed for PVA/CS 2.5 wt.% (pH = 3.65 after 21 days of storage). This finding is ascribed to the effects of respiration and the consumption of organic acids due to fruit senescence. In agreement with the above, Roopa et al. [52] found that pH changes in CS-coated Hami melons were slower than those in uncoated melons.

### 3.12. Changes in the Surface Color

**Figure 3:**
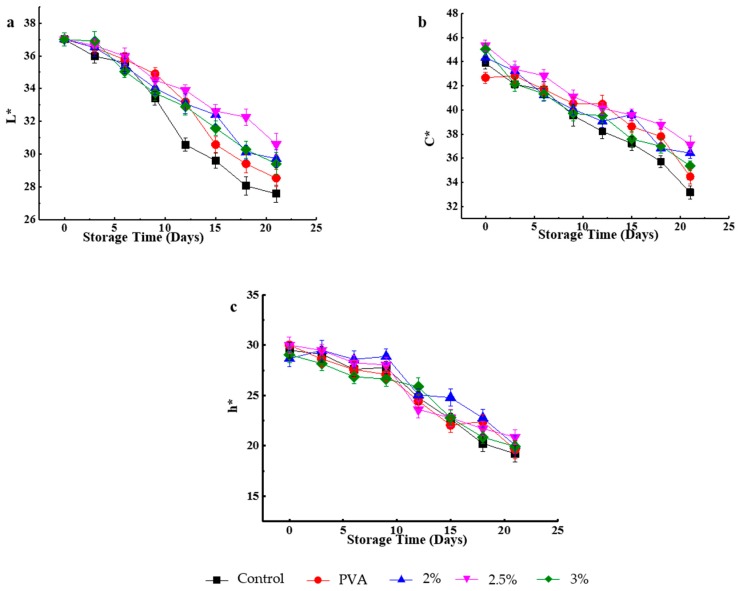
Effect of not packing and packing with PVA and different PVA/CS bilayer films on the (**a**) L*, (**b**) C*, and (**c**) h* values of strawberry during different storage times (t = 6).

Surface color changes during 21 days of storage at 5 ± 2 °C were evaluated by measuring L^*^, h^*^, and C^*^ values, and the obtained results are presented in Figure 3. Notably, the L^*^ values of all samples significantly decreased during storage, with the smallest changes observed for PVA/CS-packaged samples. The above figure reveals that no significant changes (*p* > 0.05) of L^*^ values were observed in all cases until day nine. After day nine, a significant (*p* < 0.05) decrease of L^*^ was observed for the unpackaged sample, and significant (*p* < 0.05) skin darkening was observed for the same after 21 days. In general, the h^*^ and C^*^ values of all samples decreased after 21 days of cool storage, with the largest decrease observed for unpackaged strawberries, probably because they were most affected by fungal decay and water loss. The decrease in C^*^ values indicated a shift to less vivid colors, while that of h^*^ indicated strawberry skin darkening, which is probably induced by oxidative browning reactions typical of fruit senescence. The absence of packaging, which acted as a protective barrier, accelerated the senescence of fruit, causing loss of intracellular liquid and tissue collapse [53]. Conversely, PVA/CS bilayer materials slowed down the senescence of strawberries in view of the antibacterial properties of CS, and the corresponding samples consequently featured higher values of L^*^, h^*^, and C^*^. Fruit skin color was best preserved in the case of PVA/CS 2.5 wt.% packaged samples.

### 3.13. Mathematical Modeling and Shelf Life Prediction

**Figure 4:**
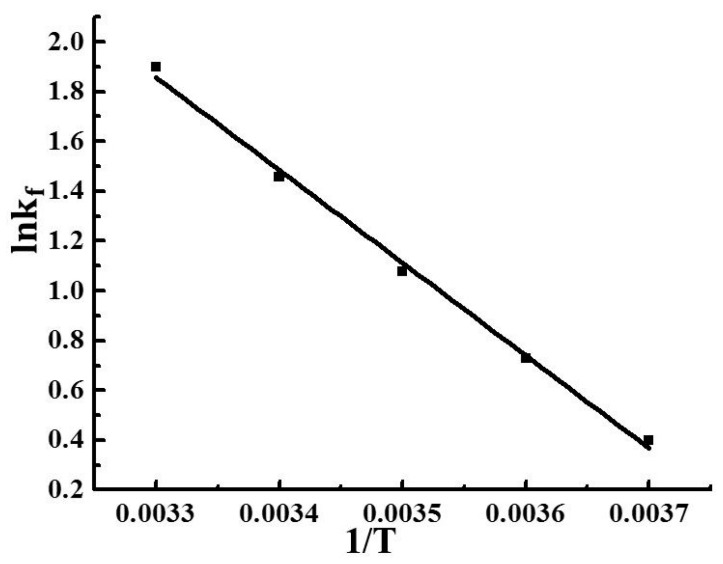
Effect of storage temperature on the index rate constant of decay index ($k_{f}$) of samples.

**Figure 5:**
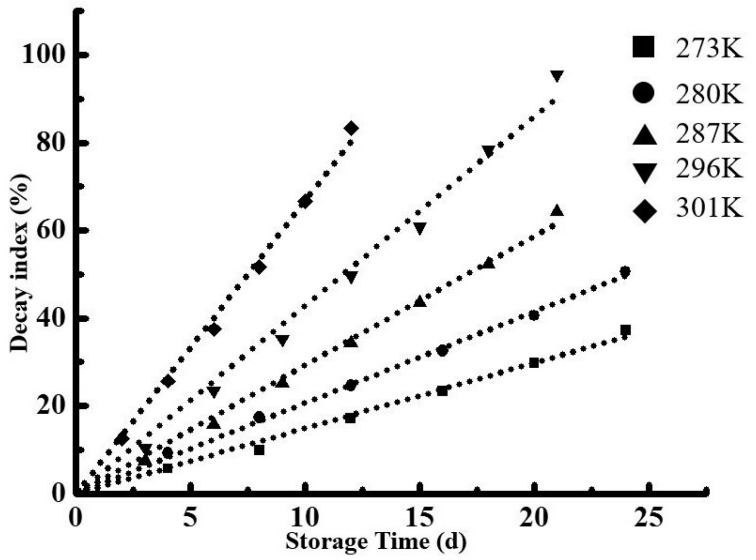
Effect of storage temperature on the index rate under different temperatures.

Previous studies demonstrated that, for most foods, the decrease in quality with time obeys zero- or first-order kinetics [54]. Specifically, zero-order kinetics is described by *A*_t_ = *A*_0_ + *kt*, while first-order kinetics is described by ln(*A*_t_) = ln(*A*_0_) + *kt*, where *A*_t_ is the quality of fruit at time *t*, *A*_0_ is the initial quality of fruit (*t* = 0), and *t* is time. Figure 4 and Figure 5, as well as Table 6, show the effect of the storage temperature on the decay index rate constant (*k*_f_) of PVA/CS 2.5 wt.% packaged strawberry fruit and the coefficients of zero- and first-order linear regressions. The sums of coefficients for zero- and first-order models were obtained as 4.9739 and 4.9418, respectively. According to Table 6, the zero-order reaction was dominant, and the fact that the corresponding regression coefficients were all greater than 0.9 implies high fitting precision. Therefore, the decay index change of PVA/CS 2.5 wt.% packaged strawberries can be best described by a zero-order model as *F*_t_ = *F*_0_ + *k*_f_*t*, where *F*_t_ is the sample decay index determined at a certain temperature and time, *F*_0_ is the initial decay index (*F*_0_ = 0), *k*_f_ is the fruit type- and temperature-dependent rate constant of decay index change, and *t* is time. As tissue deterioration is a biological process, the temperature dependence of *k*_f_ is assumed to follow the Arrhenius law, i.e., *k*_f_ = *A*exp(−*E*_a_/*RT*), ln*k*_f_ = lnA − *E*_a_/*RT*, where *R* is the universal gas constant (8.314 J·mol^−1^·K^−1^), *A* (day^−1^) represents the decay rate constant at reference temperature *T* (K), and the activation energy *E*_a_ (J·mol^−1^) expresses the dependence of the decay rate *k* on sample temperature *T* (K). Substitution of *A* = 1.404 × 10^11^ and *E*_a_ = 3.098 × 10^4^ (Figure 4) into the above equation affords *k*_f_ = *A*exp(−*E*_a_/*RT*) = 1.404 × 10^11^ × exp(−3.726 × 10^3^/*T*).

### 3.14. Model Test

**Figure 6:**
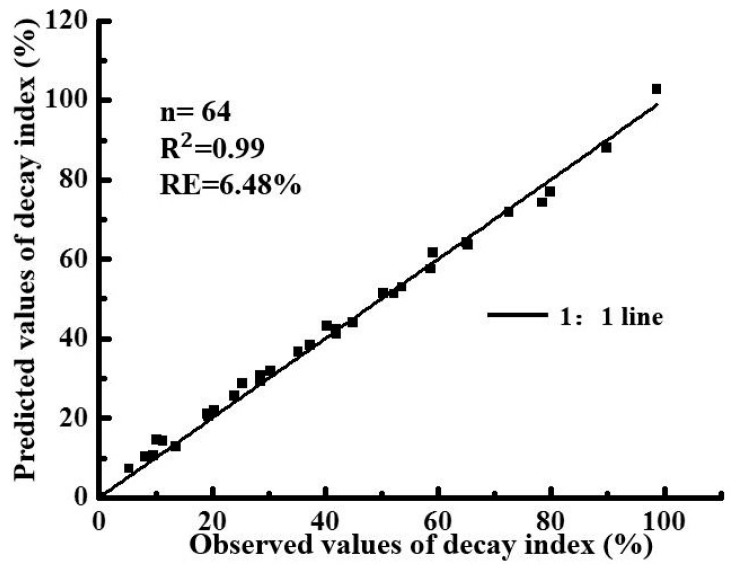
Correlation between predicted values and actual values of decay index prediction models.

Figure 6 shows that the correlation between the predicted and actual decay index values is characterized by *R*^2^ = 0.99, RE < 10%. Therefore, the developed model is concluded to be well suited for predicting the decay index of strawberries packaged in PVA/CS 2.5 wt.%.

## 4. Conclusions

Herein, PVA/CS bilayer films with varying PVA and CS contents were prepared using ultrasonication times of 0–30 min for the preparation of CS solutions. The best results were obtained using an ultrasonication time of 25 min, which allowed the biopolymer to be broken into particles small enough to form a uniform PVA/CS bilayer film with superior morphology and mechanical/antibacterial properties. During low-temperature strawberry preservation experiments, PVA/CS bilayer film packaging slowed the process of strawberry metabolism and delayed ripening, as indicated by the concomitant changes in the weight loss, firmness, TSS, TA, ascorbic acid content, decay percentage, and color. Thus, the above films could efficiently maintain the quality of strawberries throughout the low-temperature storage period, and the shelf life of fresh strawberries packaged in PVA/CS 2.5 wt.% bilayer film was determined as 21 days at 5 ± 2 °C and a relative humidity of 60 ± 5%.

## Figures and Tables

**Table 1:** Five-point system for evaluating the degree of strawberry decay.

Degree (Severity) of Decay	Area of Infected Fruit Surface (%)
1	1–2
2	21–40
3	41–60
4	61–80
5	>81 + concomitant sporulation

**Table 2:** The effect of ultrasonic treatment on film forming solution viscosity. PVA—polyvinyl alcohol; CS—chitosan.

Samples	PVA	PVA/CS 2 wt.%	PVA/CS 2.5 wt.%	PVA/CS 3 wt.%
**5 min**	3420.00 ± 35.51 A,a	3506.67 ± 25.67 A,a	4920.00 ± 26.33 B,a	10,626.67 ± 35.31 C,a
**10 min**	2386.67 ± 45.57 A,b	3693.33 ± 83.27 B,a	4626.67 ± 55.26 C,a	9440.00 ± 74.35 D,b
**15 min**	2166.67 ± 46.52 A,b	2946.67 ± 84.33 B,b	4613.33 ± 57.35 C,a	7773.33 ± 80.83 D,b,c
**20 min**	2048.00 ± 38.33 A,b	2640.00 ± 70.23 B,b	4366.67 ± 83.27 C,b	7053.33 ± 79.02 D,b
**25 min**	1993.33 ± 22.37 A,c	2453.33 ± 50.44 B,c	4235.00 ± 33.53 C,b	6926.67 ± 52.11 D,b
**30 min**	2001.00 ± 25.34 A,b	2606.67 ± 33.67 B,b	4353.33 ± 20.52 C,b	7023.33 ± 35.64 D,b

Values with the same letter are not statistically different, according to Duncan’s multiple range test at *p* < 0.05. a, b, c: mean values with the same letter in the same column are not significantly different (*p* > 0.05) (t = 6). A, B, C, D: mean values with the same letter in the same row are not significantly different (*p* > 0.05) (t = 6).

**Table 3:** The mechanical characteristics of different materials according to different ultrasound treatment times.

	PVA		PVA/CS 2 wt.%		PVA/CS 2.5 wt.%		PVA/CS 3 wt.%	
	Strain (%)	Stress (MPa)	Strain (%)	Stress (MPa)	Strain (%)	Stress (MPa)	Strain (%)	Stress (MPa)
5 min	123.23 ± 3.75 a	32.20 ± 0.28 a	98.29 ± 10.52 a	34.26 ± 2.33 a	104.68 ± 12.09 a	37.47 ± 0.67 a	87.39 ± 10.67 a	33.52 ± 1.34 a
10 min	125.49 ± 9.06 a	33.72 ± 0.09 a	101.42 ± 9.83 a	37.23 ± 1.07 a	107.79 ± 9.79 a	38.25 ± 2.07 a	96.27 ± 8.67 b	36.84 ± 2.33 a
15 min	131.47 ± 5.33 a	51.11 ± 1.03 b	105.37 ± 8.37 a	53.35 ± 2.33 b	114.43 ± 3.26 b	59.25 ± 2.52 b	105.29 ± 5.23 c	53.35 ± 0.95 b
20 min	154.52 ± 7.21 b	54.14 ± 2.36 b	121.67 ± 8.02 b	67.99 ± 0.79 c	132.24 ± 10.82 c	59.69 ± 1.65 b	107.91 ± 6.87 c	51.05 ± 0.86 b
25 min	155.29 ± 4.35 b	60.25 ± 0.72 b	124.33 ± 4.57 b	70.67 ± 0.85 c	143.15 ± 6.43 d	69.86 ± 0.82 c	118.14 ± 10.05 d	60.22 ± 1.07 c
30 min	160.37 ± 5.67 b	67.03 ± 0.97 c	126.67 ± 6.67 b	72.09 ± 0.97 c	144.21 ± 5.82 d	75.52 ± 1.54 c	120.52 ± 8.27 d	69.33 ± 2.31 d

Values with the same letter are not statistically different, according to Duncan’s multiple range test at *p* < 0.05. a, b, c: mean values with the same letter in the same row are not significantly different (*p* > 0.05) (t = 6).

**Table 4:** Oxygen permeability (OP) and water vapor permeability (WVP) values of films based on PVA as control and bilayer films based on PVA and different concentration of CS.

Samples	Oxygen Permeability (cm^2^·m^2^·day^−1^·MPa^−1^)	Water Vapor Permeation (g·cm^−1^·s^−1^·Pa^−1^)
**PVA**	0.12 ± 0.04 b	10.11 ± 2.14 b
**PVA/CS 2 wt.%**	0.15 ± 0.07 b	12.43 ± 3.72 b
**PVA/CS 2.5 wt.%**	0.16 ± 0.08 b	14.93 ± 4.09 b
**PVA/CS 3 wt.%**	0.39 ± 0.11 a	22.99 ± 5.57 a

Values indicated with the same letter are not statistically different (*p* > 0.05) (t = 6).

**Table 5:** pH analysis of strawberries treated with different films during storage.

Day	Unpackaged	PVA	PVA/CS 2 wt.%	PVA/CS 2.5 wt.%	PVA/CS 3 wt.%
**0 days**	3.45 ± 0.09 A,a	3.45 ± 0.10 A,a	3.45 ± 0.09 A,a	3.45 ± 0.08 A,a	3.45 ± 0.08 A,a
**3 days**	3.47 ± 0.13 A,a	3.48 ± 0.13 A,a	3.45 ± 0.11 A,a	3.46 ± 0.09 A,a	3.47 ± 0.10 A,a
**6 days**	3.51 ± 0.18 A,b	3.49 ± 0.17 A,a	3.47 ± 0.14 A,a	3.46 ± 0.12 B,a	3.47 ± 0.12 A,a
**9 days**	3.57 ± 0.24 A,c	3.55 ± 0.22 A,b	3.52 ± 0.22 A,b	3.50 ± 0.19 B,b	3.55 ± 0.22 A,b
**12 days**	3.64 ± 0.29 A,d	3.61 ± 0.26 A,c	3.59 ± 0.23 A,c	3.54 ± 0.20 B,b	3.59 ± 0.22 A,b
**15 days**	3.68 ± 0.36 A,d	3.69 ± 0.35 A,d	3.64 ± 0.32 A,d	3.59 ± 0.29 B,c	3.65 ± 0.31 A,c
**18 days**	3.73 ± 0.44 A,e	3.72 ± 0.41 A,d	3.69 ± 0.40 A,e	3.63 ± 0.36 B,c	3.67 ± 0.38 A,c
**21 days**	3.77 ± 0.56 A,e	3.74 ± 0.55 A,d	3.70 ± 0.51 A,e	3.65 ± 0.47 B,c	3.68 ± 0.49 B,c

Values with the same letter are not statistically different, according to Duncan’s multiple range test at *p* < 0.05. a, b, c: mean values with the same letter in the same row are not significantly different (*p* > 0.05) (t = 6). A, B, C, D: mean values with the same letter in the same row are not significantly different (*p* > 0.05) (t = 6).

**Table 6:** Coefficients of zero- and first-order linear regression.

Reaction Order	Storage Temperature					
	273	280	287	294	301	∑
**Zero Order**	0.9931	0.9982	0.9957	0.991	0.9959	4.9739
**First Order**	0.9751	0.9884	0.9874	0.994	0.9969	4.9418
